# Polymerization kinetics of 3D-printed orthodontic aligners under different UV post-curing conditions

**DOI:** 10.1186/s40510-024-00540-4

**Published:** 2024-10-28

**Authors:** Thomas Manoukakis, Alexandros K. Nikolaidis, Elisabeth A. Koulaouzidou

**Affiliations:** Division of Dental Tissues’ Pathology and Therapeutics (Basic Dental Sciences, Endodontology and Operative Dentistry), School of Dentistry, Aristotle University Thessaloniki, Thessaloniki, 54124 Greece

**Keywords:** Orthodontic, Aligners, 3D-printing, FTIR, Post-curing, Polymerization kinetics, Nitrogen

## Abstract

**Background:**

The purpose of the study was to measure the degree of conversion (DC) of direct-printed aligners (DPA) that were post-cured under ambient and nitrogen atmosphere at specific time intervals and investigate the kinetics of polymerization reaction of this material.

**Methods:**

A total of 48 aligners were produced in 4 printing series by a 3D printer with TC-85DAC resin (Graphy Inc). From each series of printing, 12 aligners were included. The aligners were divided into two groups according to their post-curing conditions. One group was post-cured under ambient air with the presence of oxygen and the other under a nitrogen atmosphere, both using the same UV post-curing unit recommended by the company. The aligners were post-cured at six different time intervals: 1, 2, 3, 5, 10, and 20 min. Each time interval included 8 aligners, with 2 aligners from each series. The DC of the cured aligners was measured by means of attenuated total reflection Fourier-transform infrared spectroscopy (ATR-FTIR) through acquisition of the respective spectra for each UV-curing condition. Statistical analysis was performed to compare the results and differences within each atmosphere post-curing protocol, as well as between the different selected atmosphere conditions. Statistical significance level was set at p-value ≤ 0.05.

**Results:**

Pairwise analysis between post-curing protocols showed statistically significant differences only at the first minute of polymerization. Post-curing with nitrogen did not yield statistically significant results across different time intervals. Post-curing in ambient air showed some significant differences on the 1st and 2nd minute of the post-curing process.

**Conclusions:**

Almost complete double bond conversion was observed. Significant differences were observed only during the first minute of polymerization under the nitrogen atmosphere.

## Introduction

Digital technologies, such as intraoral scanners, computer-aided design (CAD) software, and three-dimensional (3D) printing [1], have substantially transformed the orthodontic landscape. In particular, the utilization of 3D-printing technology in the fabrication of orthodontic aligners has gained significant attention due to its potential to enhance treatment efficiency, patient comfort, and treatment outcomes [2]. One of the key advantages of 3D-printed aligners is their accurate and precise fit as well as the replication of the patient’s dentition [2, 3]. With the ability to control their thickness in designated areas and create a unique biomechanical system [4, 5], they are designed to apply precise and controlled forces to specific teeth, gradually moving them into their desired positions over time. However, the predictability of these movements still needs to be tested in comparison to thermoformed ones [6, 7].

Although up to now, aligners have been made from materials like polyethylene terephthalate glycol (PETG) or polyurethane through thermoforming [8, 9], most 3D-printed aligners are derived from a liquid photocurable resin such as Aliphatic Vinyl Ester Urethane. This resin is initially transformed into a pliable form by a 3D-printer using stereolithography (SLA). It is centrifuged to remove excess resin and then undergoes a crucial post-curing process within specialized polymerization chambers. This stage is key to fully polymerize the aligners and endow them with their final mechanical properties, biocompatibility and aesthetic-transparency [10]. The advent of 3D-printing in orthodontics, particularly in the production of aligners, marks a significant technological advancement. Despite this progress, challenges persist in ensuring the mechanical strength, durability and biocompatibility of these 3D-printed orthodontic aligners [11].

3D-printing resins and especially resins intended for producing orthodontic aligners are toxic, irritant, and potentially allergenic before polymerization, rendering them non-biocompatible. They attain biocompatibility mainly during the 3D-printing and UV-curing stages. Inadequate polymerization could potentially lead to irritation or allergic responses in patients. When aligners are used intraorally, they are constantly exposed to triggers found in the oral environment. This might lead to the release of particles and the leaching of compounds and substances into the mouth [12, 13]. Nevertheless, recent research regarding the biological profile of the 3D-printed aligners from Pratsinis et al. [14] and studies about thermoformed aligners and adhesives [15–17], indicate that the in vitro levels of cytotoxicity and estrogenicity of these materials are not particularly alarming. It must be highlighted that these results are valid when the protocol and equipment of the company is strictly followed. This was underscored in a recent study [18] where moderate cytotoxicity was observed when post-curing was performed by a different unit than instructed. Regarding the material itself, Tera Harz TC85-DAC is likely an aliphatic vinyl ester–urethane polymer, which may be functionalized with methacrylate [19]. Unlike other thermoformed orthodontic retainer material [20], Tera Harz TC85-DAC resin showed no amounts of leached bisphenol A (BPA). However, urethane dimethacrylate (UDMA) has been detected and its effect needs to be more thoroughly examined [13].

The polymerization kinetics, involving the degree conversion (DC) of a monomer into a polymer during both the 3D-printing and the post-curing process, plays a pivotal role in determining these properties [21]. While extensive research has been conducted on polymerization in various materials, studies specifically addressing 3D-printed orthodontic aligners are markedly scarce. This gap is concerning, especially considering the increasing focus on the biocompatibility of these medical devices [11, 14, 22]. The limited research highlights the urgent need to investigate how the post-curing process influences the mechanical and biological properties of 3D-printed aligners. The post-curing phase is particularly critical for enhancing material mechanical properties, dimensional stability and transparency [23–25]. Establishing standardized protocols for this process is crucial to ensure the safety and efficacy of these devices. Innovations such as nitrogen atmosphere and high-power output in post-curing units promise to inhibit oxygen interference and achieve a higher DC, yet more data on kinetics of polymerization are needed.

This study aims to address the knowledge gap by focusing on TC85-DAC (Graphy Inc., Seoul, Korea) resin which is considered the first to receive approval from the Korean and United States Food and Drug Administration and is widely used in production of 3D-printed clear orthodontic aligners. For this purpose, the DC of this resin will be determined under different post-curing conditions, comparing ambient air and nitrogen atmospheres across different time intervals. The following null hypotheses were established; (1) there is no statistical difference between the DC achieved in a nitrogen environment and normal atmosphere when aligners are post-cured for the same selected time, (2) the increase of the post-curing time has no significant contribution to a higher cross-linking of the resin and subsequently to the obtained DC when post-cured in ambient air and (3) when post-cured in nitrogen atmosphere. This approach is expected to offer essential insights on how the post-curing environment affects the setting process and the speed of the procedure. The proposed findings could lead to more efficient and reliable manufacturing methods, optimizing mechanical properties [26] and safety of these aligners in clinical applications.

## Materials and methods

### STL file

**Fig. 1:**
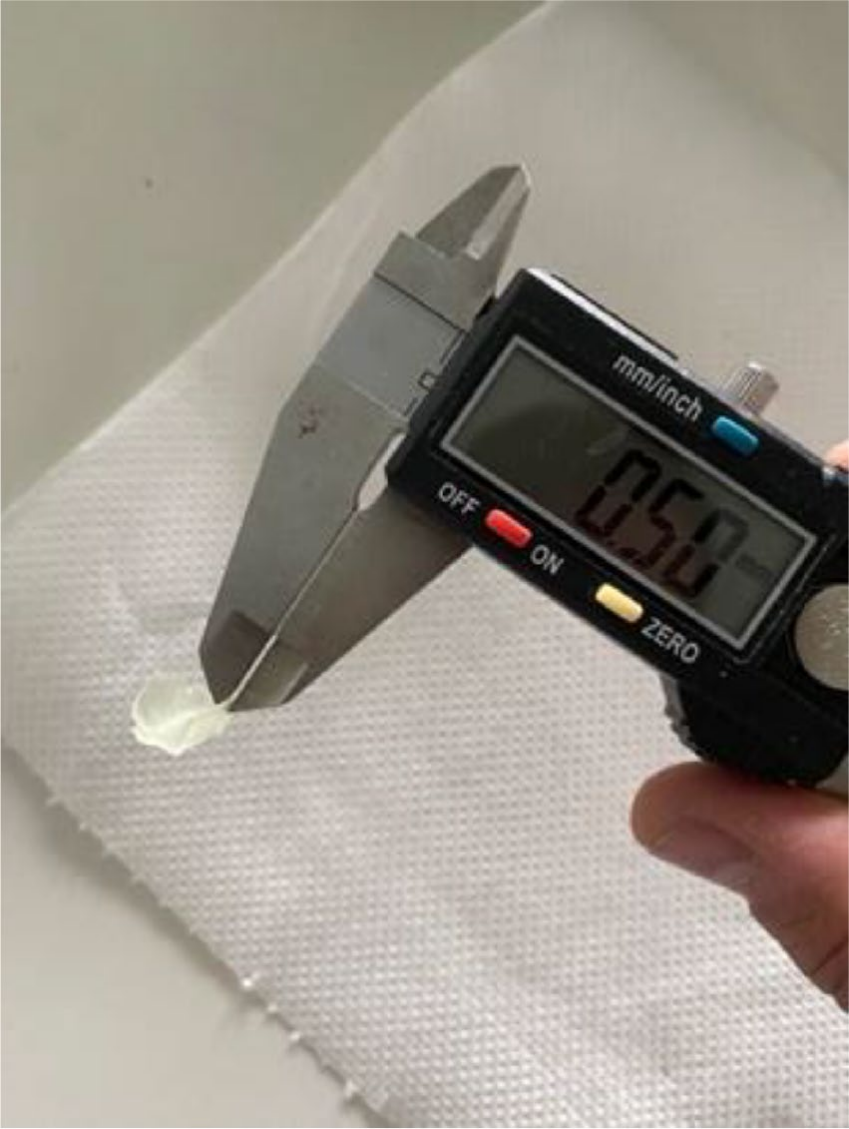
Measured thickness of the aligner (0.5 mm) coinciding with the thickness designed in the CAD software

**Fig. 2:**
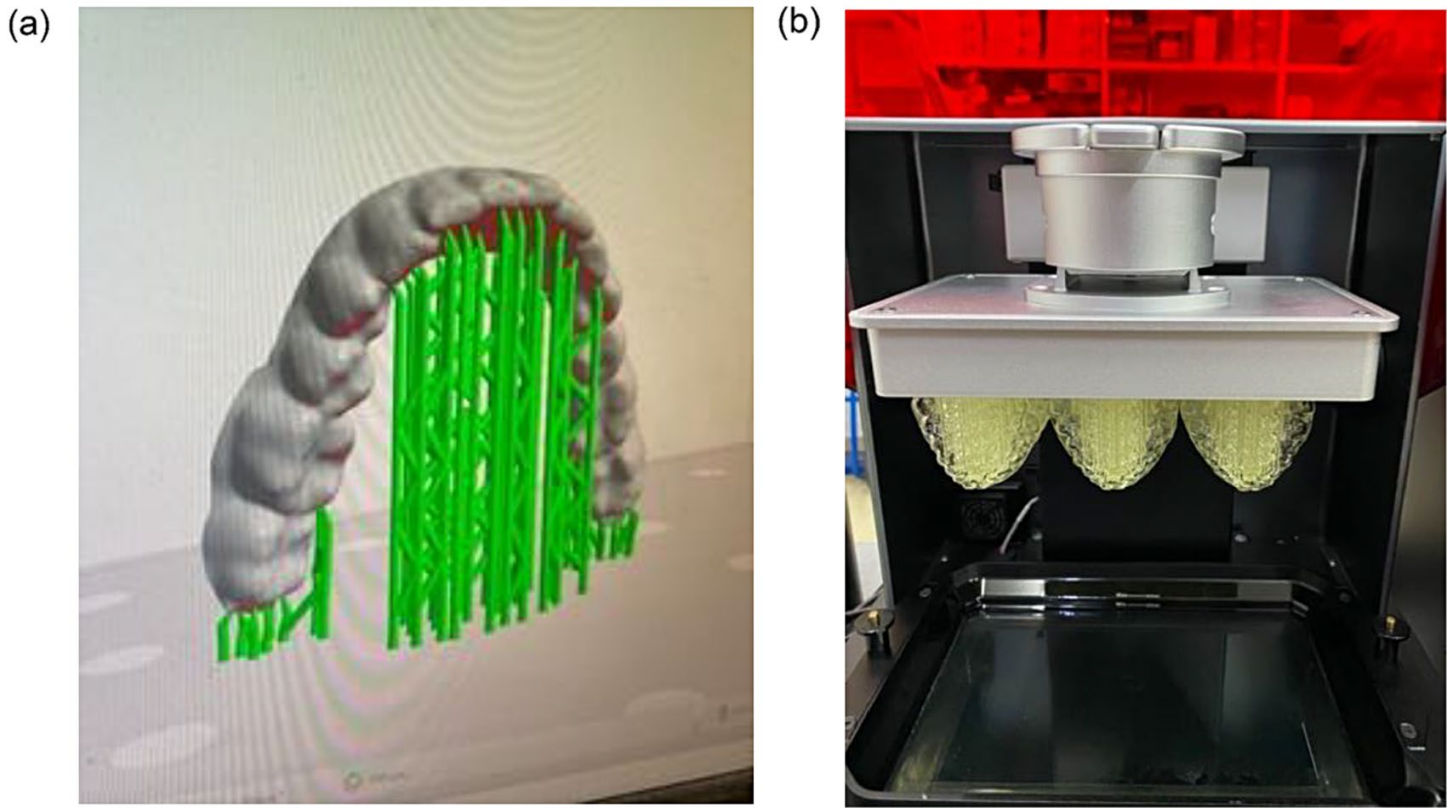
(**a**) Aligners are designed to be printed in an almost vertical orientation with a support network to ensure proper printing and dimensional stability. (**b**) After completion of the 3D-printing process the aligners are ready to be removed from the platform

A full arch model was used to create the orthodontic aligners. The produced STL file was placed almost vertically at 80 degrees and the thickness was set to 0.5 mm (Fig. 1). Supports were also placed to facilitate the printing and dimensional stability of the produced aligner (Fig. 2).

### 3D-printing of aligners

The FlashForge Focus 6 K (Zhejiang Flashforge 3D technology Co., LTD, China) 3D-printer was programmed to print the orthodontic aligner using the Tera Harz TC-85 DAC (Graphy Inc., Seoul, Republic of Korea) resin at a layer thickness of 100 μm. This setting was optimized through preliminary tests to ensure the highest print quality for the resin. After the printing process which lasted 1 h and 40 min, the aligners were processed using a centrifuge machine as recommended by the company, set at 600 rpm for 4 min, with the inner surface of the aligner facing outwards. This method ensures that any residual resin is not trapped inside the aligner. The printed aligners were then subjected to a quality check for any defects such as warping or layer separation. Four printed series were produced, containing a total of 48 aligners, namely 24 for ambient air conditions and 24 for nitrogen atmosphere at the subsequent post-curing process. Regarding the distribution of the aligners, 12 aligners were included from each series of printing. Half of them in each series were destined for nitrogen curing and the others for ambient conditions. Each time frame required 8 aligners, 2 from each series. Therefore, for the 1-minute time interval a total of 8 aligners were needed from 4 series of printing. Four of them were then post-cured in ambient air conditions and the other 4 in nitrogen air. Same procedure was followed for the rest of time intervals, in order to consider differences associated with the 3D-printing procedure.

### Post-curing process

Upon successful printing, each aligner underwent a post-curing process using the curing unit Tera Harz Cure THC2, (Graphy Inc., Seoul, Korea) equipped with an integrated nitrogen generator. Prior to each curing cycle, a consistent nitrogen atmosphere was ensured by filling the chamber with the inert gas. Aligners were placed horizontally into the chamber and exposed to UV light (405 nm light-emitting diode-LED-wavelength, 1000 mW/ cm^2^ UV irradiance) at the following time intervals: 1, 2, 3, 5, 10, and 20 min. These intervals were selected with a focus in the early stages of the post-curing process. Since the protocol suggested by the company has evolved together with the development of new units, studies in the past have utilized different post-curing times. The above setting parameters were also repeated under ambient air conditions.

### Degree of conversion (DC%) measurements

To control sampling consistency, a small segment was carefully excised from the molar area of each aligner using a precision cutting tool. The post-cured at different time intervals and the 3D-printed samples (*n* = 4) were analyzed by means of attenuated total reflection Fourier transform infrared (ATR-FTIR) spectrometer (Cary 630, Agilent Technologies, USA). Spectra acquisition was performed at the 4000 –650 cm^-1^ scanning range, using 4 cm^-1^ resolution and a total of 32 scans. Reference spectra of the uncured resin were also recorded. The net peak areas of the total C = C stretching vibrations (1634 and 1620 cm^-1^) and the N-H bending absorptions (1523 cm^-1^) were calculated using the tangent baseline technique. The degree of monomer conversion (DC%), which expresses the percent amount of the C = C which reacted at each period time, was assessed based on the following formula:

$$\,DC\,\% = \left( {1 - {{{{\left( {{{{A_{c = c}}} \over {{A_{N - H}}}}} \right)}_{cured}}} \over {{{\left( {{{{A_{c = c}}} \over {{A_{N - H}}}}} \right)}_{uncured}}}}} \right) \times 100$$

### Statistical analysis

Descriptive statistics were calculated using mean values and standard deviation (SD). The assumption of normal distribution was investigated for the bias using the Kolmogorov-Smirnov test based on Monte Carlo statistical method (*p* = 0.952), followed by variance homogeneity tests. Due to lack of previous knowledge relative to polymerization kinetics of this material, a priori Power Analysis was performed with software G* Power Version 3.1.9.4 [27] and it was based on the results of a pilot study. For effect size d = 3, significance level a = 0.05 and power = 0.80 the minimum required sample size for each type of atmosphere within each time interval was estimated *n* = 4 aligners and a total of 48 aligners was used. Univariate analysis of variance (UNIANOVA) was used to evaluate the association of degree of conversion (DC) with the type of atmosphere (ambient air or nitrogen) and the duration of the post-curing process. Bonferroni corrections were made to adjust for multiple tests. Statistical analysis was performed using «IBM SPSS Statistics 28». Statistical significance level was set at p-value ≤ 0.05.

## Results

**Fig. 3:**
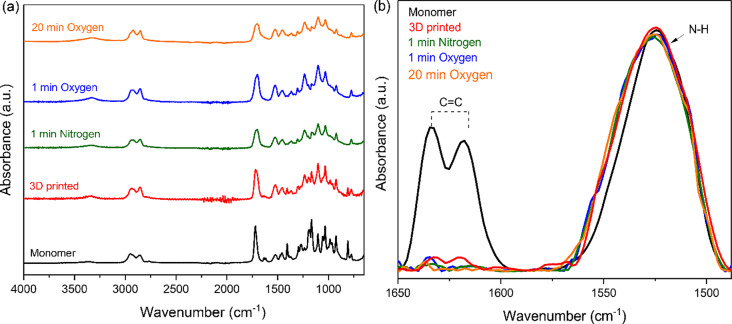
(**a**) ATR-FTIR spectra acquired for the unset Tera Harz TC-85 DAC, the cured resin after a 3D-printing process, and the post-cured aligner under different atmosphere conditions at specific time intervals. (**b**) Absorption bands corresponding to C = C stretching (1634 and 1620 cm^-1^) and N-H bending vibrations (1523 cm^-1^), used to calculate the percent degree of conversion (DC %) for uncured material, 3D-printed and different post-cured aligners

Representative FTIR spectra recorded for the studied unset resin, 3D-printed, and post-cured aligners are presented in Fig. 3a. The broad peak at 3371 cm^-1^ due to N-H stretching vibrations and the secondary amine absorption band at 1523 cm^-1^ correspond to the possible presence of urethane functional groups [19]. The strong peaks at 2953 & 2857 cm^-1^ are attributed to aliphatic C-H symmetric and asymmetric stretching vibrations respectively. A very strong peak at 1720 cm^-1^ is assigned to C = O stretching, while the double absorption bands 1634 and 1620 cm^-1^ are due to aliphatic C = C stretching vibrations. The absorption peaks correspond to the major functional groups of the methacrylate structure of the initial monomer. Additional peak assignments: ν_s_(O-C-O): 1475 cm^-1^, δ_as_(C-H): 1457 cm^-1^, and δ_s_(C-H): 1405, 1392 cm^-1^, γ_s_(C-CH_3_): 1370 cm^-1^, ν_s_(C-O): 1231 cm^-1^, δ_out of plane_ (C-C-O): 1192 cm^-1^, ν_as_(C-O): 1166 cm^-1^, δ_out of plane_ (O-C-O): 1100 cm^-1^, ν(C-N): 1031 cm^-1^, δ_out of plane_(C-H): 984, 926 cm^-1^, δ_out of plane_ (CH_2_-CH-O): 810 cm^-1^, where ν: stretching, δ: bending, γ: wagging vibrations.

Figure 3b illustrates typical FTIR spectra in the narrow range of 1650 –1490 cm^-1^ used to determine the degree of double bond conversion for the tested materials based on the peak areas due to the C = C and N-H absorptions. The high intensity peaks occurred at 1523 cm^-1^ (N-H bands) were considered as internal reference standard, almost retaining the same pattern in comparison to C = C bands at 1634 and 1620 cm^-1^. The latter peaks were significantly lowered for the 3D-printed and the post-cured resins. In particular, the intensity of the absorption band at 1634 cm^-1^ was gradually decreased when turning from the monomer to the 3D-printed, and finally to the UV irradiated resins under distinct conditions. The secondary peak at 1620 cm^-1^ was clearly attenuated for the post-cured products implying a high contribution to the decrease of the A_C=C_/A_N−H_ peak area ratio which favors the increment of the calculated DC values.

The tests of between-subjects effects, used to determine whether there are any significant differences between the studied groups on the dependent variable, showed that 65.5% (R^2^ = 0.655) of the DC variances can be justified by the “post-curing duration” (*p* < 0.001), “post-curing atmosphere conditions” (*p* = 0.029), and “post-curing atmosphere conditions *duration” (*p* = 0.065) groups. The effect size measured by the partial eta squared revealed that the influence of the “duration” (H^2^ = 0.589) group was higher than the “atmosphere conditions” (H^2^ = 0.125) and the “atmosphere conditions *duration” (H^2^ = 0.242) groups respectively.

The mean value of the DC% after the completion of the 3D-printing process was determined to 63.22% based on 1000 bootstrap samples (std error = 2.651, lower value = 59.16%, upper value = 68.62%, 95% confidence interval).

In terms of the oxygen atmosphere conditions applied during the post-curing process, the obtained DC% values increased from 94.41 to 97.44% over the range of 1–20 min (Table 1). More specifically, after 1 min UV-curing the DC% was significantly lower than after 3 min (*p* = 0.010), 5 min (*p* < 0.001), 10 min (*p* < 0.001), and 20 min (*p* < 0.001) respectively. Moreover, the DC% value calculated at 2 min was significantly lower than after 20 min irradiation (*p* = 0.035). No other statistically significant differences were found within the rest groups (*p* < 0.05).

**Table 1:** Degree of conversion values (DC%) determined at diverse time intervals under oxygen post-curing conditions

Oxygen	
Post-curing duration (min)	Mean (SD)
1	94.41 (1.38)
2	95.86 (0.62)
3	96.20 (0.31)
5	96.78 (0.46)
10	97.04 (0.51)
20	97.44 (0.48)

The 1 min had significant difference with 3, 5, 10, 20 min time intervals and that the 2 min had a significant difference only with the last (20 min) time interval

When the UV chamber was conditioned with nitrogen gas, the achieved DC% was 96.26% just after 1-minute photocuring and reached the ultimate value of 97.74% after 20 min (Table 2). There were no significant differences between the studied groups (*p* < 0.05).

**Table 2:** Degree of conversion values (DC%) determined at diverse time intervals under nitrogen post-curing conditions

Nitrogen	
Post-curing duration (min)	Mean (SD)
1	96.26 (0.64)
2	96.35 (0.66)
3	96.48 (0.41)
5	96.78 (0.31)
10	96.82 (0.76)
20	97.74 (0.89)

**Fig. 4:**
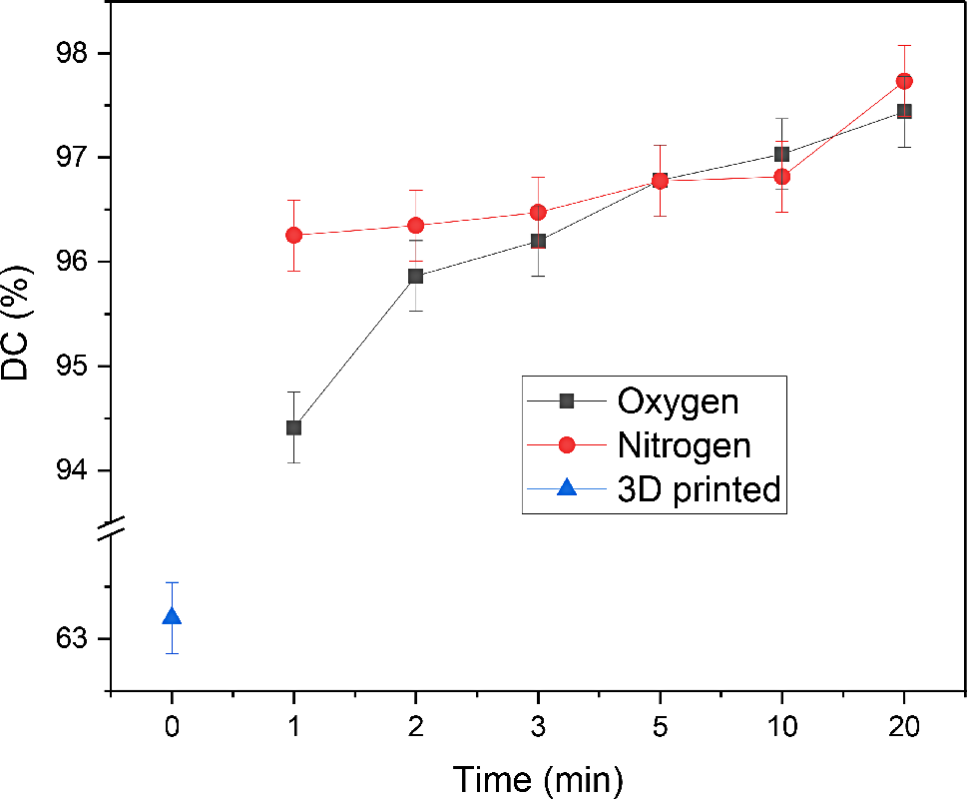
Degree of conversion vs. setting time of 3D-printed resin and set resins under different post-curing conditions

Comparative plots of DC% changes over time for the 3D-printed and UV-cured aligners under different atmosphere conditions are demonstrated in Fig. 4. It is obvious that both post-curing procedures have a strong effect on the conversion value of the 3D-printed resin at the early stage of polymerization (1 min). Table 3 lists the p-values derived from the multiple comparisons of DC% mean values between oxygen and nitrogen atmosphere at several time intervals of the post-curing process. A strong inhibition effect of oxygen on the free radical polymerization mechanism was observed at the early step of the UV-curing (1 min), as it was revealed by the significantly lower DC% value (*p* < 0.001) than the obtained conversion under inert atmosphere. No other statistical differences were detected within the rest groups (*p* < 0.05), although the polymerization efficacy under nitrogen remained superior until 5 min setting (Fig. 4), when the DC% value reached the same level for both selected conditions (*p* = 0.988). Further irradiation of aligners resulted in elevated conversions characterized by slight fluctuations discriminated at 10 min (*p* = 0.654) and 20 min (*p* = 0.543).

**Table 3:** Pairwise analysis between different post-curing atmosphere conditions at constant setting duration

Post-curing duration (minutes)	Oxygen	Nitrogen
	*p*-values	
1	**< 0.001**	
2	0.320	
3	0.571	
5	0.988	
10	0.654	
20	0.543	

Values marked in bold have a statistically significant difference (*p* < 0.05)

**Fig. 5:**
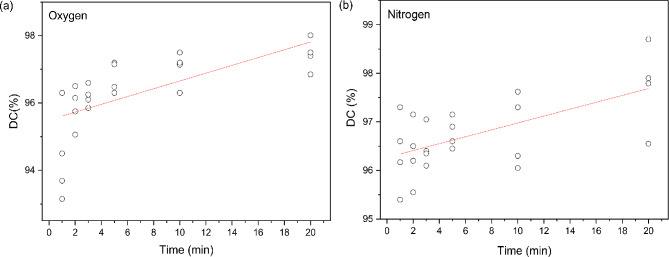
Fitting lines to scatter plots of DC% data vs. post-curing duration drawn for (**a**) oxygen and (**b**) nitrogen atmosphere, using unweighted linear regression method. Mean values of the measurements were used and the fitting lines were drawn accordingly

Scatter plots of DC% values against setting time, involving a fitted smooth curve through points on basis of an unweighted regression model, are presented in Fig. 5 for both the applied oxygen and nitrogen atmosphere during post-curing procedure. The Pearson correlation coefficient calculated for oxygen (*r* = 0.652327 > 0.60) and nitrogen (*r* = 0.620196 > 0.60) atmosphere exhibits linear correlation between data for both conditions. This model allows the DC calculations for oxygen (y = 0.12x + 95.5) and nitrogen (y = 0.07x + 96.27) atmosphere over the range of 5–20 min where better linearity between variables has occurred. Moreover, the slope of the linear equation shows that the increased tendency of DC over post-curing duration is more intensive by operating the chamber saturated with oxygen gas.

**Fig. 6:**
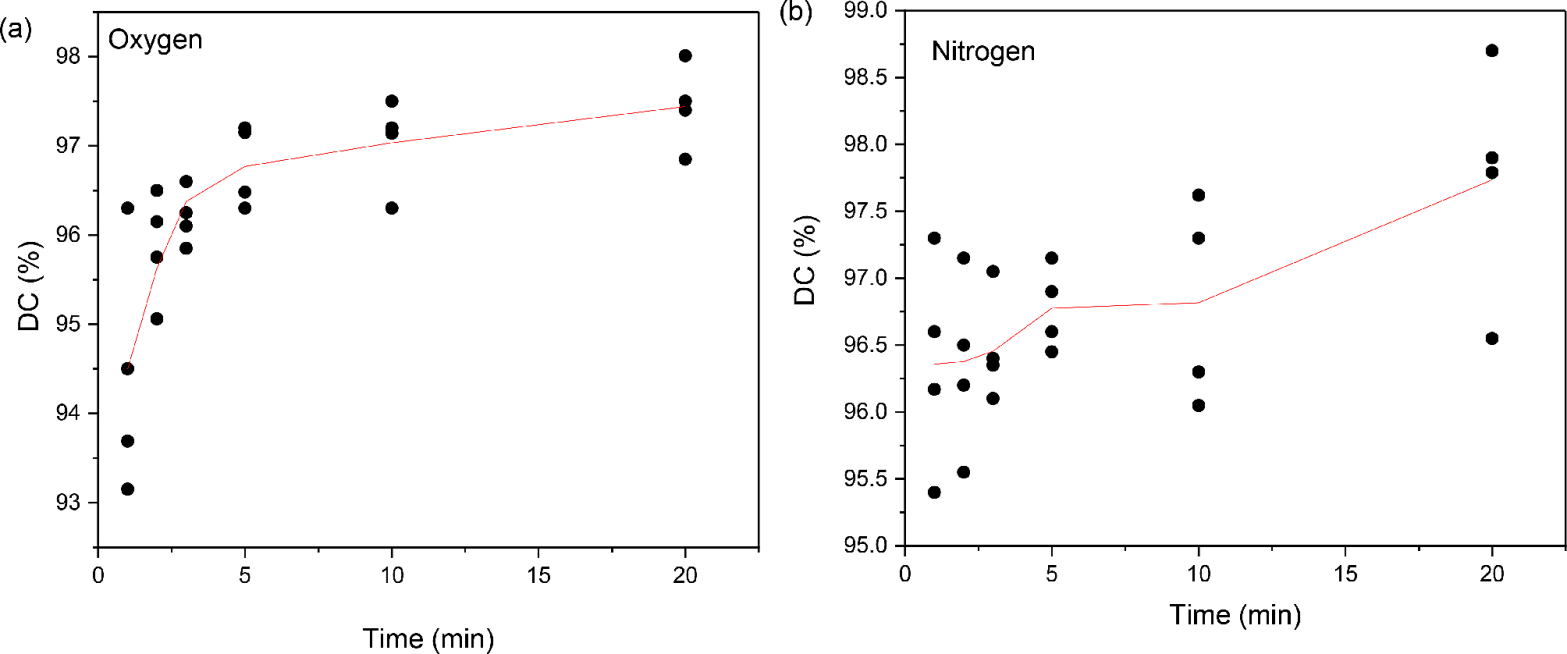
LOESS curve fitting (local polynomial regression) model used for the correlation of DC% data with UV-irradiation time under (**a**) oxygen and (**b**) nitrogen atmosphere

Best fitting lines were also plotted by means of the locally estimated scatterplot smoothing (LOESS) method (Fig. 6). Regarding the oxygen conditions (Fig. 6a), an early abrupt increase of the double bond conversion is observed for the first 3 min of irradiation, followed by a lower curing rate up to 5 min. This incremental trend seems to be less effective between 5 and 20 min. Under nitrogen feeding of the UV-chamber (Fig. 6b), a short plateau of the DC values occurs during the initial 3 min of the setting process. Moreover, an intensive augmentation of the polymerization rate is detected over 3–5 min, while the conversion degree remains constant within 5–10 min. Afterwards, the curing rate presents a linear increasing pattern until 20 min irradiation.

## Discussion

According to the acquired FTIR spectra of this study, the double covalent bonds(C = C) peaks corresponding to methacrylic groups of the unset and 3D-printed material were significantly attenuated on set groups, almost in all time intervals, indicating an extended conversion of double bonds regardless the ultimate UV-curing conditions used.

Based on the obtained results the first null hypothesis should be rejected, as it was revealed that for the first minute of UV-polymerization there are statistically significant differences on the DC values between the different atmosphere conditions tested, although on the rest of the time intervals there were no statistical significance observed. The observed changes in polymerization behavior come in accordance with the recent results published by Mattle et al. [10]. Another study by Wada et al. [26, 28] noticed that the use of nitrogen UV post-curing conditions in combination with a liquid crystal display (LCD) printer achieved the highest DC however was studying a different resin for production of 3D-printed occlusal splints. In this experiment in order to achieve the same DC with post-curing in ambient air, the time needed was 3 times more than in nitrogen atmosphere. The steep augmentation of DC achieved under nitrogen conditions at the first minute of UV polymerization (96.26%) could be beneficial, considering that the whole process becomes time saving and efficient.

The DC measurements under ambient atmosphere showed that there is statistical significance between 1-minute post-curing time and the selected 3, 5, 10 and 20 min post-curing periods, thus we can reject the second null hypothesis for the aforementioned studied groups. The DC value achieved after 1-minute post-curing was found to be 94.41%, namely higher than the DC (83%) observed for Tera Harz TC-85DAC aligner resin in a previous study [13] by means of Cure M machine after 24 min curing under ambient air conditions. Graphy initially introduced the UV curing device Cure M, tailored for aligners. Subsequent investigations by the company, however, suggested that the aligners’ qualities were not optimal [22], and the more advanced Tera Harz 2 UV-curing device was utilized for the same purpose. This recently introduced machine includes a nitrogen generator that connects to a high-pressure air-line, infusing nitrogen into the device’s curing chamber. Also based on the company’s technical characteristics, LED power output of the new device is dramatically increased; from 80 W to 200 W, therefore promising better polymerization of the 3D-printed aligner which is addressed in this research. As a result, the higher LED intensity of the UV device can lead to remarkable DC values allowing lower levels of monomer remnants in the surface of the aligner and ensuring a more complete polymerization process [18, 34, 35]. Lastly, no statistical significance was observed between time intervals of UV post-curing in nitrogen conditions. Therefore, the third null hypothesis of the study can be accepted regarding the applied inert UV-curing environment. According to FTIR findings, the effect of nitrogen atmosphere is crucial especially in the initial stages of the polymerization and not the whole procedure. In particular, immediately after the initial 3D-printing, the presence of nitrogen in combination with the high intensity output strongly favours the free-radical polymerization at the first studied stage of UV-curing, thus reflecting almost full double bond conversion (DC = 96.26%). Once the UV chamber is filled with nitrogen, the generated stream displaces the oxygen levels which inhibit the polymerization through the formation of less reactive peroxy radicals (ROO·) [29]. This means that at this stage, the added advantage of a nitrogen atmosphere could contribute to a more uniform polymerization, as nitrogen curing can reduce oxygen inhibition, leading to improved surface quality and consistency. Additionally, uniform polymerization can enhance mechanical properties such as strength, durability, and fatigue resistance, reducing the risk of deformation or breakage. More complete curing could possibly lead to less residual monomers, reducing allergic reactions and irritation, thereby improving biocompatibility and patient safety. Enhanced transparency and prevention of discoloration over time are also significant aesthetic advantages. Understanding the polymerization kinetics of 3D-printed aligners allows clinicians to make informed decisions on selection and handling, improving treatment safety and efficiency. Consistent material performance minimizes the risk of aligner failure, enhancing patient satisfaction and treatment outcomes. Given the complexity of these materials compared to thermoformed ones, it is crucial to strictly follow the fabrication protocols recommended by the manufacturer while deepening our understanding of their properties.

As far as limitations of the study are concerned, the present study does not consider the temperature of the UV post-curing chamber as a contributing factor in enhancing the final product of the polymerization and affecting the DC as observed in another study [30]. The quality of the 3D-aligner file at the time of export is a critical factor that can significantly contribute to defects in aligners or printing failures. As stated by Panayi [22], there can be variability in the quality of aligner files even when they are generated from the same software. Furthermore, the use of different software programs can result in 3D-aligner files of varying quality for the same patient [31]. Issues such as corrupted files, defects in the aligner mesh, or other problems may result in structural issues with the aligner or lead to printing failures. Other crucial parameters, commonly referred to as “irradiant exposure conditions”, encompass aspects like power and the time or speed of exposure, and these might contribute to variations in printing results [32–34]. Every printer employs a distinct technology to polymerize the resin, utilizing either a laser beam, a light projector, or LEDs. In the study by Zinelis et al. [35], 3D-printers using LCD technology yielded aligners with increased mechanical properties, such as hardness, which are of considerable clinical importance. However, despite these findings, there is currently scarce evidence to suggest that these significant mechanical properties are associated with the polymerization of the aligners and clinical effectiveness of orthodontic treatments. In the framework of the present study, the effect of the post-curing conditions on the DC of the obtained aligners was investigated to improve control of the UV-curing process. Further studies involving mechanical tests of the aligners, produced at specific DC values, after long-term aging in simulated oral conditions, could provide useful data regarding their long-term stability and wear resistance. Lastly, this study was performed using a specific type of an LCD 3D printer, in conditions resembling those found in a laboratory and not in the premises of the company, following the protocols known at that time. It is expected that continuous improvements will be implemented in all aspects of the procedure in the future.

## Conclusions

Under the limitations of this study, it was found that the TC85-DAC resin could attain polymerization degrees surpassing previously recorded levels, thus imparting promising attributes for aspects like mechanical properties, biocompatibility and the appearance of the aligner. Additionally, it seems that the use of a nitrogen generator with this specific post-curing unit had a significant effect on the DC of the aligners only during the first minute of the post-curing process and not afterward. This increase in polymerization efficiency might be linked to the curing unit particularly to its higher output along with other factors. The presence of oxygen should be further investigated to determine whether it is associated with factors other than DC, such as the mechanical properties and biocompatibility of the 3D-printed orthodontic aligners. The presented results may contribute to the establishment of novel practices in the fabrication of orthodontic aligners and the duration of the post-curing process.

## Data Availability

The authors make the data from this study accessible upon reasonable request.

## Abbreviations

ATR-FTIR: Attenuated total reflection fourier-transform infrared spectroscopy RE-AIM
BPA: Bisphenol A
CAD: Computer-aided design
DC: Degree of double bond conversion
DPA: Direct-printed aligners
LCD: Liquid crystal display
LED: Light-emitting diode
SD: Standard deviation
SLA: Stereolithography
STL: Standard tessellation language
UDMA: Urethane dimethacrylate
UNIANOVA: Univariate analysis of variance
